# In Vitro Bioaccessibility of Bioactive Compounds from Rosehip-Enriched Corn Extrudates

**DOI:** 10.3390/molecules27061972

**Published:** 2022-03-18

**Authors:** Marta Igual, Adriana Păucean, Dan Cristian Vodnar, Purificación García-Segovia, Javier Martínez-Monzó, Maria Simona Chiş

**Affiliations:** 1 Food Investigation and Innovation Group, Food Technology Department, Universitat Politècnica de València, Camino de Vera s/n, 46022 Valencia, Spain; marigra@upvnet.upv.es (M.I.); pugarse@tal.upv.es (P.G.-S.); xmartine@tal.upv.es (J.M.-M.); 2 Department of Food Engineering, Faculty of Food Science and Technology, University of Agricultural Sciences and Veterinary Medicine of Cluj-Napoca, 3-5 Mănăştur Street, 400372 Cluj-Napoca, Romania; adriana.paucean@usamvcluj.ro (A.P.); 3 Institute of Life Sciences, Faculty of Food Science and Technology, University of Agricultural Sciences and Veterinary Medicine of Cluj-Napoca, 3-5 Mănăştur Street, 400372 Cluj-Napoca, Romania; dan.vodnar@usamvcluj.ro (D.C.V.)

**Keywords:** *Rosa canina* powder, corn extrudates, in vitro digestibility, bioaccessibility

## Abstract

The rosehip (*Rosa canina* L.) fruit has gained researchers′ attention due to its rich chemical composition in vitamin C, phenols, carotenoids, and high antioxidant activity; meanwhile, polymers such as pea protein are generally recognized as exhibiting a protection role against the extrusion process. Corn snacks extrudates obtained by replacing corn flour with 10% *R. canina* powder (R) and 10% *R. canina* with pea protein (RPP) were evaluated for the physicochemical, textural, optical, and nutritional characteristics. A sample manufactured without *R. canina* powder was used as a control. Hardness, crispiness, chewiness, and solubility index (WSI) of the final extrudates were improved by addition of *R. canina* and pea protein powder (PP); meanwhile, b* (yellow/blue coordinate), *C* (chroma), and h* (tone) optical parameters were significantly different from the control sample (*p* < 0.05). Extrusion highlighted a negative impact on total phenols, carotenoids, vitamin C, and antioxidant activity extrudates, while PP exhibited a good protection against the extrusion process. In vitro digestion increased the bioaccessibility of vitamin C, folate, antioxidant activity, total phenols, and total carotenoids mainly on RPP extrudates.

## 1. Introduction

The lockdown caused by the COVID-19 pandemic situation has changed lifestyle and consumers’ behaviors worldwide, increasing snacking tendencies [1]. Recently, during the third wave of the COVID-19 pandemic in Spain, which started in 19 February 2021, Spanish consumers′ habits changed, and snacking increased, mainly due to perceived pandemic stress. This led to a 36.1% consumption increase of snacks, nuts, jellybeans, and soft drinks [2]. Likewise, during lockdown in Italy, the consumption of “comfort foods”, such as snacks, chips, and chocolate, increased [3]; meanwhile, in Denmark and Germany, the consumption of fresh food significantly dropped. In the UK, the sale value for snacks, crisps, and nuts, being considered staple foods, is estimated to reach £4.8 billion in 2022, consumed by 90% of households [4]. In line with this, recently, Ciudad-Mulero et al. highlighted that snack-type product consumption increased, being mainly consumed by children, and probably could represent a real source for improving consumption health [5].

From a nutritional viewpoint, snack foods are considered nutritionally poor, as they are rich in fat, sugar, and salt [6]. Expanded snacks are mainly manufactured using extrusion technology, with the main ingredient being corn flour [7]. Corn is the cereal with the highest worldwide production [8] and is texturally an optimum raw material for snack manufacturing [7]. The textural properties of cereals are mainly based on their expansion characteristics and improvement of starch digestibility through a gelatinization process [9]. Nutritionally, corn is claimed as the “poor man’s nutricereal” [10] and does not meet the needs of health-conscious consumers [11].

Therefore, there is a market niche for healthy snacks that could be considered vectors for biologically active compounds leading to functional food. Thus, rosehip (*Rosa canina*) could be a sustainable and nutritional alternative that could enriched corn extrudates. Rosehip (*Rosa canina* L.) is defined as a shrub from the *Rosaceae* family that can grow in loamy and clay soils with a high coppice shoot ability, used for improving soil properties [12]. It is widespread worldwide, from Western and Central Asia to Europe, Russia, Pakistan, the Caucasus region, Iran, Armenia, Iraq, and Kashmir, and it is one of the revenue-generating species in rural development projects [13]. It has a rich nutritional content in bioactive compounds such as vitamin C, carotenoids, polyphenols, folate, fatty acids, and minerals, exhibiting high antioxidant activity [1].

Several in vitro studies have highlighted the biological and pro-healthy properties of rosehip extract. For instance, Fattahi et al. [14] showed that rosehip extract plays a key role in the development of pancreatic β-cell line, with a positive role in the diabetes mellitus disease. Moreover, recently, Bahrami et al. [15] proved that a novel oligosaccharide from *Rosa canina* fruits could be considered as a reliable anti-diabetic agent due to its ability to increase insulin production, whilst Lattanzio et al. [16] showed that rosehip extract could act as a tool in the management of inflammatory diseases. In line with this, Turan et al. [17] showed that *Rosa canina* could be considered as a potential ingredient in the development of an natural anticancer product, and mentioned that *Rosa canina* ethanolic extract has a potential effect on human breast and colon cancer lines and could act against human cervix cancer.

During an extrusion process, due to high temperature and pressure, some of the bioactive compounds might be affected negatively or even lost. To protect the bioactive compounds in biopolymers, such as resistant maltodextrin, maltodextrin, and betacyclodextrin, pea protein powder (PP) could be used. In our recent study [9], we demonstrated that biopolymers, such as resistant maltodextrin, maltodextrin, beta-cyclodextrin, and pea protein powder (PP), could be successfully used to protect bioactive compounds against the extrusion process. From the aforementioned biopolymers, during the extrusion process, PP exhibited the highest protection of phenolic compounds, carotenoids, vitamin C, and folate. The nutritional quality of food is directly correlated with the bioaccessibility of the bioactive compounds, which become available in the small intestine through digestion for further absorption [18]. To the best of our knowledge, this is the first study investigating the in vitro digestibility of corn extrudates enriched with *R. canina* powder (R) and PP.

Therefore, the aim of this study was to investigate the corn extrudates′ bioactive compounds’ bioaccessibility using the in vitro gastrointestinal digestion model. To achieve this goal, the corn flour was enriched with 10% R, and PP was used as a biopolymer. A sample without PP was used as a control. Furthermore, corn extrudates’ functional values, physicochemical characteristics, and nutritional properties were evaluated.

## 2. Results

### 2.1. Physical, Textural, and Optical Properties of Mixtures and Corn Extrudates

Table 1 shows the mean values of water loss (W_L_), specific mechanical energy (SME), melt pressure (P), and barrel temperatures (T_1_ and T_2_), respectively. In the present study, the SME ranged from 1099 to 1309 J/g, the highest value being scored by the rosehip mixture (RM) sample. Samples with rosehip powder with or without pea protein lost significantly higher water content than the control (*p* < 0.05), while the addition of R and RPP increased the P value and barrel temperatures, as displayed in Table 1.

The physicochemical characteristics of extrudates are displayed in Table 2. The addition of R and RPP mainly influenced the water absorption index (WAI), water solubility index (WSI), hygroscopicity (Hy), expansion index (SEI), crispness work (Wc), spatial frequency of structural ruptures (N_sr_), and number of peaks (N_o_) characteristics of the final extrudates. For instance, WAI values ranged between 3.12 and 5.19, Hy values scored between 23.0 and 26.3, and N_sr_ values ranged between 10.15 and 14.2 mm^−1^. With respect to the water content (x_w_), samples ranged from 3.45 g_w_/100 g to 1.70 g_w_/100 g for the CE sample and RE sample, respectively.

Table 3 shows the mean values and standard deviations of color coordinates and total color differences between the corn mixtures and extrudates. Adding R and RPP caused a significant decrease of L* (luminosity) in the mixtures (*p* < 0.05). However, this effect was not observed in the extrudates. The differences in the rest of the coordinates due to adding R and RPP were the same in mixtures and extrudates, showing an increase of a* (red/green coordinate), b* (yellow/blue coordinate), and C* (chroma), and a decrease of h*. The extrusion process decreased the values of all color parameters except h* (tone). After extrusion, the value of h* was maintained regarding mixtures of RPPE, but increased in CE and RE. Total color differences due to extrusion (ΔE2) were higher in CE than RE and RPPE samples. Total color differences between the enriched samples and control (ΔE1) were higher in mixtures.

**Figure 1:**
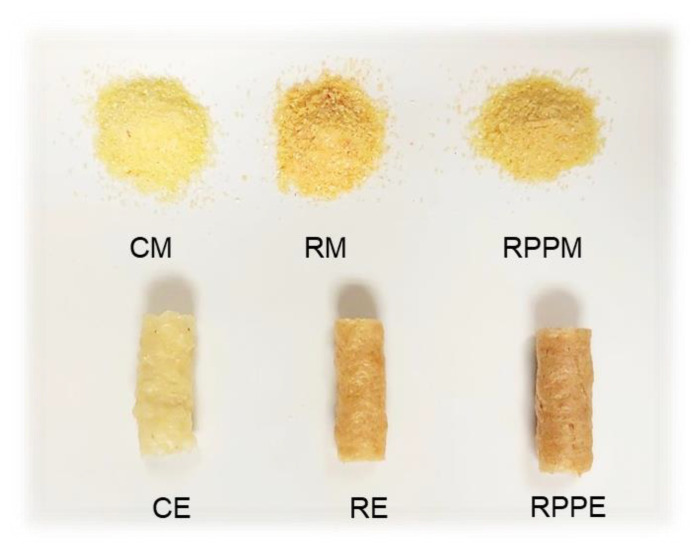
Appearance of studied mixtures (M) and extrudates (E). C: control, R: rosehip enrichment, and RPP: rosehip + pea protein enrichment.

In Figure 1, the most obvious color differences can be observed in the mixtures, especially the reddest tonality of RM (Table 3). The appearance of the extruded samples enriched with R or RPP (Figure 1) was apparently very similar to the control samples, and therefore might be considered visually acceptable.

### 2.2. Nutritional Characteristics of Mixtures and Corn Extrudates

Table 4 displays the bioactive compounds from mixtures and extrudates. RM and RPPM showed significantly higher values for all the bioactive compounds studied compared to CM (*p* < 0.05). Likewise, RE and RPPE presented significantly higher values of the bioactive compounds studied than CE (*p* < 0.05). On the other side, PP addition increased ascorbic acid (AA) and vitamin C (VC) levels (Table 4).

After extrusion, the values of bioactive compounds decreased significantly in all studied samples (*p* < 0.05) (Table 4). However, a protective effect can be observed in bioactive compounds of PP because losses of FT, TC, TP, and AC were lower in RPPE than RE samples. For instance, as displayed in Table 4, FT RE extrusion loss was 45.5%, while RPPE FT content registered a percentage of extrusion loss of 16.58%. With respect to the RE sample, the TC content reached a value of 7.85 mg/100 g_dry weight_, whereas RPPE samples registered a value of 5.75 mg/100 g_dry weight_, with initial values in mixtures of 43.74 mg/100 g_dry weight_ and 22.73 mg/100 g_dry weight_, respectively. Furthermore, the RE TP amount decreased during extrusion by 49.43%, whereas the RPPE samples saw a decrease of 37.75% after extrusion, emphasizing the protective effect of PP on TP content.

### 2.3. Nutritional Characteristics of Extrudates after In Vitro Extrusion

Table 5 shows the bioactive compounds content of mixtures and extrudates after in vitro digestion. With respect to mixtures, addition of R and RPP influenced the mixtures’ content in a positive way, and therefore acted similarly on those of the extrudates. For instance, after in vitro digestion the mixtures, the TP increased from 6 mg/100 g_d.w._ to values of 180.6 mg/100 g_d.w._ and 155 mg/100 g_d.w._, for RM and RPPM samples, respectively. Moreover, regarding the TF extrudates’ content after in vitro digestibility, RE and RPPE samples scored enhanced values of 132 mg/100 g_d.w._ and 129 mg/100 g_d.w._, respectively. For VC content, increased values were registered in mixtures and extrudates and in vitro digestion (Table 5).

Considering the AC mixtures’ and extrudates’ bioaccessibility, the biggest AC bioaccessibility was highlighted by RM samples, while the lowest was reached by CM samples. Extrudates exhibited similar values for RE and RPPE values, whilst CE showed the smallest bioaccessibility amount.

**Figure 2:**
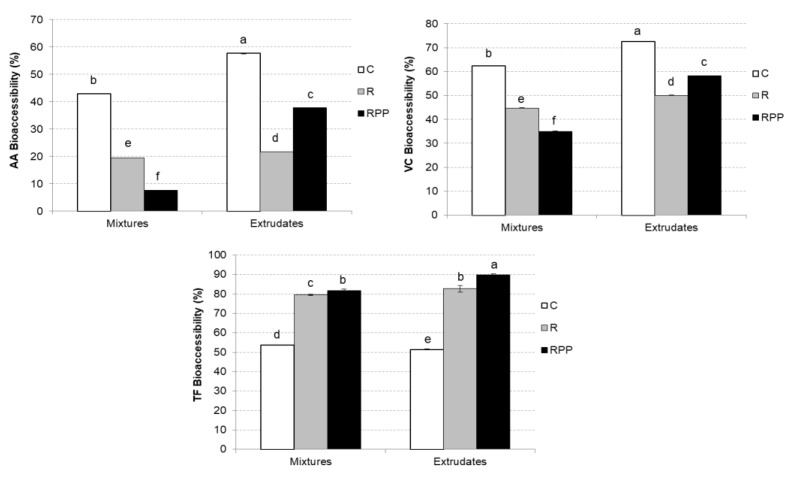
Mean values and standard deviation of ascorbic acid (AA), vitamin C (VC), and total folate’s (TF) bioaccessibility. Letters indicate homogeneous groups established using ANOVA (*p* < 0.05).

Figure 2 displays the AA, VC, and TF bioaccessibility of mixtures and extrudates after the simulated in vitro digestibility. Briefly, with respect to AA bioaccessibility, extrudates exhibited higher values than mixtures, except from CM bioaccessibility. On the other hand, VC and TF bioaccessibility content was significantly higher (*p* < 0.05) in RPPE samples, showing values of 59% and 90%, respectively.

**Figure 3:**
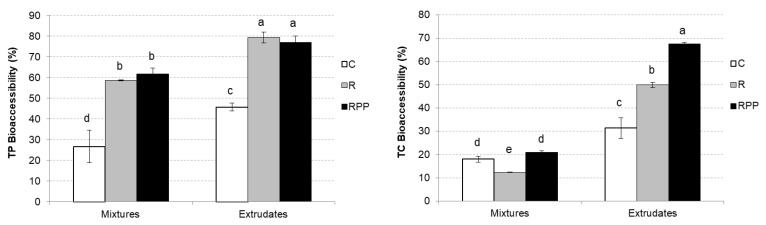
Mean values and standard deviation of total phenol’s (TP) and total carotenoid’s (TC) bioaccessibility. Letters indicate homogeneous groups established using ANOVA (*p* < 0.05).

Mean values and standard deviation of TP and TC mixtures’ and extrudates’ bioaccessibility is displayed in Figure 3. TP extrudates emphasized significant differences (*p* < 0.05) compared to mixtures, while the same trend was observed in TC extrudates. For example, TC mixtures’ bioaccessibility ranged in values of 11–21%, while extrudates’ values ranged from 31% to 68%.

Extrusion increased the bioaccessibility of TP and TC significantly (*p* < 0.05). Furthermore, R and RPP addition increased the bioaccessibility of TP both mixtures and extrudates significantly. However, R and RPP addition only increased significantly the bioaccessibility of TC in extrudates. AA bioaccessibility presented the same trend as VC bioaccessibility; AA and VC were significantly more bioaccessible in extrudates than in mixtures (*p* < 0.05). However, R and RPP addition significantly decreased AA and VC bioaccessibility in mixtures and extrudates (*p* < 0.05). The values of TF bioaccessibility ranged from 50% to 90% (Figure 3). Remarkably, R and RPP addition increased TF bioaccessibility significantly in both mixtures and extrudates (*p* < 0.05).

## 3. Discussion

The extrusion cooking technique is a multifunctional process where the raw materials are subjected to different parameters such as temperature, pressure, and shear forces with paramount impact on the biochemical extrudates’ composition, together with protein denaturation, starch degradation, and the Maillard reaction [19]. Barrel temperatures (T_1_ and T_2_), melt pressure (P), water loss (WL), and specific mechanical energy (SME) are the main parameters of the extrusion process. There are several factors that can affect the SME values. For instance, moisture content, particle size, and composition of the material used for extrusion, whilst SME was reported to evaluate the suitability of extruder machines for product development, according to [20]. The variation of SME during the extrusion process was also highlighted in our previous study [1] where we valorized rosehip by-product (*Rosa canina* L.) in corn extrudates. This variation could be explained by the R addition and could be corelated with the water loss samples, which were biggest in the RM and RPPM samples, and then in the control (CM).

Additionally, in samples with R addition, the content of water that could be absorbed by the R fiber is probably higher; therefore, the water loss and pressure at the open of the die values will increase [1]. The higher water loss content in enriched extrudates was also mentioned by [1,10] in corn extrudates enriched with lucerne (*Medicago sativa* L.) and rosehip by-product, respectively.

According to Karkle et al. [21], the W_L_ during extrusion is strongly associated with pressure in air cells and with the extensibility characteristics of the mass, which are associated with the connection to water [21]. Thus, in samples with R or RPP, as there is an increase in fiber, there is a greater amount of water absorbed by this component, and the loss of water will be greater at the die opening due to the difference in pressure. Therefore, expansion will be smaller, as besides the higher W_L_, insoluble fibers are also biopolymers, which are already slightly extendable, unlike starch [21]. Moreover, in this study, RM and RPPM caused significantly higher pressure during the extrusion than the CM (*p* < 0.05), and pressure often increases with decreasing moisture content [22]. In this study, the water content of CM (8.5%) was significantly higher than RM and RPPM (7.9–7.6% of xw, respectively) (*p* < 0.05). This was reflected in a significantly lower pressure during extrusion in CM (*p* < 0.05). T1 and T2 showed the same trend as P, as extrusion of RM and RPPM presented significantly higher temperature than the CM (*p* < 0.05).

Extrusion produces very characteristic food structures. The physicochemical properties that can characterize these peculiar food structures to a greater extent are density, porosity, expansion index, and mechanical properties. Other interesting physicochemical characteristics for this product are those related to the product′s response to water, such as the absorption and water solubility indexes, hygroscopicity, and swelling. The values of these parameters for CE were in line with other works that studied corn-extruded products [10,23,24].

Using R and RPP modified the physicochemical properties compared to the control. However, the changes observed led to positive physicochemical improvements. Adding R and RPP significantly improved the mechanical properties of extrudates (*p* < 0.05), reducing the hardness (Fs and F_p_) and increasing the crispiness (N_sr_ and N_0_) compared to the control. Samples with R and RPP were easier to chew (W_c_) and more breakable. Incorporating R and RPP also significantly reduced the water content (x_w_) of the extrudates and the solubility index (WSI) (*p* < 0.05), providing greater stability to the product. WSI exhibits the interaction of extrudates with water [25] and emphasizes the molecular damage that can occur during extrusion due to the water-solubilized components’ releasing [26]. Incorporating R and RPP in the extrudates formulation led to a significant increase in the water absorption capacity of the extrudates compared to the control (*p* < 0.05). This can be observed in the higher WAI and SWE values (Table 2), where it is likely the fiber in R increased the absorption capacity of RE and RPPE. RPPE showed the highest values of WAI and SWE, probably because of the hydration of the protein structure but without significant differences with RE (*p* > 0.05). RE and RPPE were more hygroscopic than CE, and only RE showed significant differences with CE (*p* < 0.05). Despite the improvements found when adding R or RPP, a significant decrease was observed in the expansion and density of the extrudates at the exit of the extruder nozzle (*p* < 0.05) (Table 2). However, the porosity of the three samples was similar and within the typical ranges of corn extrudates [23,24,27]. ε values of RPPE were significantly lower than the other samples (*p* < 0.05), but the differences were very small.

To establish relationships between the parameters in Table 2, Pearson correlations were performed. x_w_ was significantly and positively related to W_c_ (0.9530, *p* < 0.05), ρ_b_ (0.8383, *p* < 0.05), F_s_ (0.8256, *p* < 0.05), and F_p_ (0.7888, *p* < 0.05); however, it was negatively related to N_0_ (0.9851, *p* < 0.05) and N_sr_ (0.9596, *p* < 0.05). When extrudates contained higher water content, snacks were denser, harder, and less crunchy. WAI was significantly and positively correlated to Hy (0.9741, *p* < 0.05) and SWE (0.9250, *p* < 0.05), and negatively correlated to WSI (0.9875, *p* < 0.05) and SEI (0.8230, *p* < 0.05). Therefore, the extrudates with higher absorption capacity presented higher hygroscopicity and lower solubility capacity in water, as well as lower expansion. As observed in the values in Table 2, snacks with R presented higher WAI values. The fiber in the rosehip implied the extrudates absorbed a greater amount of water and during extrusion suffered less expansion. This behavior was observed in other studies on corn extrudates enriched with rosehip encapsulates and lucerne [9,10]. Moreover, in line with our previous studies [1,9,24], a significant positive Pearson correlation was established between SEI and ε (0.9951, *p* < 0.05).

Regarding the color parameters, as illustrated in Figure 1 and Table 3, the addition of R and RPP caused changes in both mixtures’ and extrudates’ samples. This could be justified mainly by the chemical composition of *Rosa canina* powder, which, as we previously mentioned, is a rich source in carotenoids, from which carotenes are considered plants’ and fruits’ natural red and/or orange colorants [1]. Moreover, extrusion caused changes in the total mixtures’ color (ΔE), decreasing L*, a*, b*, c*, and h* parameters. The results are in line with those of Dogan et al. [28], who mentioned that extrusion caused darker final products with intensive red and yellow colors.

With respect to the bioactive compounds, addition of R and RPP improved both the mixtures’ and the extrudates’ nutritional characteristics. This could be justified by the rosehip chemical composition, mainly rich in vitamin C, folates, carotenoids, and phenolic compounds, as highlighted in our previous studies [1,9]. As highlighted in our recent study, *Rosa canina* powder is rich in carotenoids lycopene (78.95 µg/g), β-carotene (58.25 µg/g), and zea-ester (44.54 µg/g); furthermore, p-Coumaric acid (18.06 µg/g) and ferulic acid (9.03 µg/g) were the most important phenolic compounds [9]. On the other hand, in PP samples, AA and VC levels increased, supporting the idea that polymers during different thermal treatments could exhibit a protector effect on the vitamin and flavonoid contents [29].

Regarding the extrusion process, we could assert that it had a negative influence on the bioactive compounds. Our results are supported by a large body of literature [1,10,29]. Likewise, Anton et al. [30] showed that extrusion registered a negative impact on the phenolic acid content of corn extrudates due to their polymerization with tannins, affecting their extractability and, therefore, their antioxidant activity. Likewise, Pasqualone et al. [31] mentioned a decrease of phenolic acids due to the high temperature reached during the extrusion process. Moreover, Corrales-Bañuelos et al. [32] showed thermal processing and alkaline conditions could enhance the carotenoid degradation through isomerization and oxidation.

The significant highest correlation of bioactive compound with AC was TC-AC (0.9855, *p* < 0.05), followed by TP-AC (0.8180, *p* < 0.05). A relationship between antioxidant activity and TC was also highlighted by Cueto et al. [33], who mentioned that during the cooking process, thanks to the Maillard reaction, some bioactive compounds with antioxidant activity could be formed, which might protect carotenoids from oxidative degradation. However, a strong relationship between polyphenols and antioxidant activity was mentioned by Brennan et al. [34], who showed that extrusion process could undergo the decarboxylation of phenolic acids, leading to a decrease in extractability and antioxidant activity. Likewise, Leonard et al. [35] observed a trend between antioxidant activity and the phenolic content of extrudates, showing that the antioxidant activity of extrudates is strictly corelated with Maillard reaction bioactive compounds and phenolic acid degradation. Salazar Lopez et al. [36] also showed a significant correlation of 0.735 and 0.915 between the TP of sorghum bran extrudate and DPPH, and TP and TEAC values, respectively.

The nutrition labeling for foodstuffs from the Council directive published on 24 September 1990 [37] stated the recommended daily allowance of folate is 200 μg. Regarding this and regulation no. 1924/2006 of the European Parliament and of the Council of 20 December 2006 [38] on nutrition and health claims made in foods, extrudates enriched with rosehip are a food “high in folate.” According to the folate content shown in Table 4, the consumption of 40 and 52 g of RPPE and RE, respectively, could ensure the recommended daily folate consumption. Furthermore, it is important to mention the extrudates VC content, which has a recommended allowance of 60 mg/day. Therefore, RPPE and RE could also be considered a food “high in vitamin C” (Table 4).

The in vitro digestion analysis reproduces the chemical–enzymatic catalysis that occurs in the proximal tract of the monogastric digestive system [39]. Mean values (and standard deviation) of in vitro digestion of CM, RM, and RPPM were 78.9 (1.3), 73.6 (0.3), and 73.6 (1.5), respectively. For each formulation, a significant (*p* < 0.05) decrease in in vitro digestion % was observed after extrusion. The values of in vitro digestion % for CE, RE, and RPPE were 75.8 (0.8), 71.36 (0.07), and 70.56 (0.14), respectively. This could be explained by the fact that extrusion process induces the formation of resistant starch that physiologically behaves like dietary fiber [40]. This is in line with Gulzar et al. [41], who showed that extrusion process caused significant changes of rice flour digestibility, mainly by increasing the resistant starch content.

Therefore, the matrix loses digestibility after extrusion and more residues are carried to the large intestine. On the other side, Ye et al. [42] stated that starch gelatinization increased during extrusion, but it depends on several factors such as starch size, degree of crystallinity, degree of polymerization, non-starch components and their interactions with starch, and the amylose/amylopectin ratio. Furthermore, the addition of R and RPP significantly decreased the in vitro digestion % in both mixtures and extrudates (*p* < 0.05), probably due to the fiber content of rosehip. Moreover, the reduction in starch digestibility during the extrusion process was explained through the fiber content of the raw materials able to entrap starch granules within the protein–fiber and starch network. The gelatinization of starch could be restricted due to water availability as a main consequence of soluble non-starch polysaccharide hydration [43].

The bioaccessibility is defined as the degree of a nutrient or of a bioactive molecule dissolution from the food matrix to the gastrointestinal tract into a suitable manner to be absorbed by the organism [44,45].

Folate is defined as the generic descriptor for folic acid, and has paramount importance for developing neural tubes, prevention of birth defects during pregnancy, cardiovascular diseases, and lung carcinogenesis [46,47]. It was reported that the folic acid increased its bioaccessibility by using mesoporous silica particles [47] leading to a controlled release after consumption. Moreover, the folic acid bioaccessibility is influenced by the gastric pH [48]. For example, in cereal-based baby foods, at pH 1.5, the bioaccessibility of folic acid decrease by 68%, whereas a pH of 4 decreased the folic acid bioaccessibility by 41%.

The extrusion effect on the bioaccessibility on phenols and antioxidant activity is still controversial. For instance, brown rice and oat extrudates showed the most bioaccessible phenolics and antioxidant compounds, whereas oat extrudates showed a lesser phenol and antioxidant bioaccessibility [49]. Cereals’ bioaccessibility is strictly related to their cereal matrix and to free and bound phenolics’ sensitivity [49]. However, another study investigated the bioaccessibility of pasta manufactured with fruits, such as raspberries and boysenberries, and concluded that the addition of fruits positively influences the bioaccessibility of polyphenols [43]. Our findings agree with those who mentioned food processing could increase the phenolic compounds of pulses’ bioaccessibility [43], supporting the idea that processing could negatively influence the phenolic compounds while promoting their bioaccessibility.

In the present study, after in vitro digestion, mixtures showed a higher bioactive compounds’ content and antioxidant activity, compared with extrudates. Herrera-Cazares et al. [18] showed the extrusion process could lead to an improvement of the phenolic acid permeability at the final level of intestinal incubation, thanks to a controlled release from their bound forms. Likewise, Yagci et al. [50] showed that after in vitro digestion, total phenolic content and antioxidant activity of tomato pomace-enriched samples’ extrudates were improved, probably due to enzymatic hydrolysis during digestion, which led to a better release of the bounded compounds. Moreover, defatted rice bran registered a phenolic bioaccessibility after gastrointestinal digestion of 63.9%, whereas extruded defatted rice bran registered a value of 65.9%, showing that extrusion can improve phenolic bioaccessibility [42].

During digestion, due to the acidic conditions in the gastric phase, glycosidic bonds could also be broken, leading to an improvement in phenolic bonds, according to Liu et al. [51]. The idea that the breakdown of the glycosidic bonds during digestion is mainly caused by the acidic pH it is also supported by Yu et al. [52]. However, proteins from R and corn flour could be enzymatically hydrolyzed during the intestinal phase, leading to enhanced peptides and an amino acid content able to bind polyphenols [50]. Likewise, Melini et al. [45] stated the release of phenolic compounds during in vitro digestion could be enhanced by the breakdown of protein complexes, sugars, fiber residues, low pH, and enzymatic activity.

## 4. Materials and Methods

### 4.1. Raw Materials and Reagents

Corn grits (C) were purchased from Maicerías Españolas S.L. (Valencia, Spain). Rosehip fruits were manually harvested in October 2020 from Aldehuela (Teruel, Spain). Pea protein powder (Nutralys^®^ S85F) (PP) was supplied by Roquette S.L. (Valencia, Spain). Folic, ascorbic, and gallic acid standards were purchased from Sigma-Aldrich Inc. (Sydney, NSW, Australia); hexane, acetone, ethanol, acetonitrile, acetic acid, 2,2-diphenyl-1-picryl-hydrazyl-hydrate (DPPH), and Folin–Ciocalteu reagent were purchased from Merck (Merck KGaA Darmstadt, Germany). β-carotene was obtained from Fluka-Biochemika (Fisher Scientific S.L., Madrid, Spain).

### 4.2. Rosehip Powder Manufacturing

The rosehip, approximately 1000 g of sample, was washed and mixed by using a Thermomix (TM 21, Vorwerk, Valencia, Spain), under the following parameters: 1 min, 5200 rpm, as described in our previous study [1]. Afterward, distilled water was added (1000 g) under continuous homogenization at the same speed for 5 min. A sieve was used (light of mesh diameter 1 mm, Cisa 029077, 1 series) to filter the obtained mixture, aiming to obtain the final rosehip puree. Then, 10 g of PP was added to 90 g of rosehip puree. Then, the rosehip puree with PP and the control puree (without PP) were freeze-dried. A puree layer was placed on a standardized aluminum plate and was frozen at −45 °C (Vertical Freezer, CVF450/45, Ing. Climas, Barcelona, Spain) for 24 h before being dried in a Lioalfa-6 Lyophyliser (Telstar, Spain) at 2600 Pa and −55.6 °C for 48 h. In order to obtain a free-flowing powder, the freeze-dried samples were ground in a grinder (Minimoka, Taurus, Lleida, Spain). The powdered products are referred to as rosehip (R) and rosehip pea protein (RPP).

### 4.3. Extrusion Process and Sample Manufacturing

Extrusion was performed by using a single-screw laboratory extruder (Kompakt extruder KE 19/25; Brabender, Duisburg, Germany) with a barrel diameter of 19 mm and a length:diameter ratio of 25:1, under the following parameters: 3:1 compression ratio, a feed rate range of 3.51 kg/h with a dosing speed of 18 rpm, and a 3 mm nozzle diameter, as described in our previous work [1]. The screw rotation was 150 rpm, and the temperatures barrel sections were as follows: 25, 70, 170, and 175 °C, respectively. Extruder Winext software (Brabender, Duisburg, Germany) was used to register the barrel temperatures (T_1_ and T_2_), screw speed, motor torque, and melt pressure (P). For extruder feeding, corn grits (C) were mixed with 10% R and 10% RPP in order to obtain the rosehip mixture (RM) and rosehip and pea protein mixture (RPPM). A control sample without R and RPP addition was used as the control mixture (CM). Afterward, the mixtures were introduced in the extruder, cooled at ambient temperature, and packed in plastic bags for further analysis. The extrudates were encoded as follows: control extrudate (CE), extrudates with rosehip enrichment (RE), and extrudates with rosehip + pea protein enrichment (RPPE).

### 4.4. Physico-Chemical Mixtures’ and Extrudates’ Analysis

#### 4.4.1. Physical, Textural, and Optical Properties of Mixtures and Corn Extrudates

##### Water Activity, Content, Hygroscopicity, and Expansion Index (SEI)

Water content (x_w_) (g water/100 g sample) was analyzed according to the [53] AOAC method, while water activity (a_w_) was performed by using AquaLab PRE LabFerrer equipment (Pullman, WA, USA), according to our previous work [10]. Hygroscopicity was calculated as g of water gained per 100 g dry solids, according to Cai and Corke, 2000 [54]. Briefly, 0.5 g of the sample was placed in petri dish at a temperature of 25 °C using an airtight plastic container with Na_2_SO_4_-saturated solution with a relative humidity of 81% and maintained for 7 days. The expansion index was measured through an electronic Vernier caliper (Comecta S.A., Abrera, Spain), according to the method described by [9].

##### Porosity (*ε*), Bulk Density Extrudates, and Water Loss

Porosity is defined as the percentage of the air volume related to the total volume and calculated considering the real and bulk densities, as described by García-Segovia et al. [20]. With respect to bulk density, a helium pycnometer (AccPyc 1330, Micromeritics, Norcross, GA, USA) was used, as mentioned by García-Segovia et al. [24]. Water loss (W_L_) was calculated as the difference between the mixtures’ water content and extrudates’ water content, as described by Ribeiro et al. [23].

##### Water Absorption Index, Water Solubility Index, Swelling Index, and Specific Mechanical Energy

Water absorption (WAI), water solubility (WSI), swelling (SWE) indices, and specific mechanical energy were performed according to the method described by Uribe-Wandurraga et al. [27]. Briefly, extrudates were milled to a final particle size of 180–250 μm, and 2.5 g of each sample was dispersed in distilled water and stirred for 30 min with a magnetic stirrer, and the obtained dispersions were centrifugated at 3000× *g* for 10 min. The resulting sediment and the dissolved solid content for the supernatant were weighed and used for the calculation of WAI and WSI indices. SWE was performed using the bed volume technique, as described by Robertson et al. [55].

SME can be defined as the energy required for the production of 1 g of extrudate [56], and, according to [57], is calculated as the ratio between the torque multiplied by the screw speed (rad s^−1^), divided by the mass flow rate (g s^−1^).

##### Extrudates’ Texture Parameters

The puncture test was used for the extrudates’ texture parameters with a 2 mm cylinder diameter and a crosshead speed of 0.6 mm, performed on a TA-XT2 Texture Analyzer (Stable Micro Systems Ltd., Godalming, UK), equipped with a software (Texture Exponent, version 6.1.12.0) as described by our research group [1]. Parameters such as crispness work (W_c_), average puncturing force (Fp), spatial frequency of structural ruptures (Nsr), specific force average of structural ruptures (Fs), and peak numbers were calculated according to our previous work [58].

##### Optical Properties

CIE*L*a*b* color coordinates were measured considering standard light source D65 and a standard observer 10° (Minolta spectrophotometer CM-3600d, Konica Minolta, Chiyoda, Japan), as previously described by [1]. Measurements were taken six times for the mixtures and extruded samples. Previously, extrudates were measured on white and black backgrounds to consider translucency, and it was concluded that extrudates were not translucent. The total color differences cause by rosehip powder in mixtures or extrudates (ΔE1) were calculated. To evaluate the mixtures’ color changes due to the extrusion process, total color difference (ΔE2) was calculated between each mixture and extrudate.

#### 4.4.2. Nutritional Characteristics of Mixtures and Corn Extrudates and In Vitro Digestion

##### Ascorbic Acid and Vitamin C Content

Vitamin C was quantified as the sum of ascorbic (AA) and dehydroascorbic acids extracted through ultrasound-assisted extraction, as described by [1]. Briefly, 0.5 g of each sample was homogenized in an aqueous solution with 8% acetic acid and 3 mL of 3% H_3_PO_4_ and sonicated using an Elmasonic E15H (Elma, Singen, Germany) bath for 30 min. Afterwards, the obtained solution was centrifuged for 10 min at 400× *g* in an Eppendorf 5804 centrifuge (Eppendorf, Germany, Hamburg). The supernatant was filtered and 20 µL was injected into the HPLC-DAD-ESI-MS system equipped with a quaternary pump, autosampler, DAD detector, and coupled to an MS-detector single-quadrupole Agilent 6110 (Agilent-Technologies). An XDB C18 Eclipse column (4.5 × 150 mm) with a flow rate of 0.5 mL/min was used for separation and identification of the compounds, with 1% formic acid:acetonitrile (95:5) in distilled water (*v*/*v*) (A) and 1% formic acid in acetonitrile (B) as binary gradients. For MS fragmentation, a scanning range of 100–600 *m*/*z* in the ESI (+) mode was conducted with a capillary voltage of 3000 V, a temperature of 300 °C, and a nitrogen flow rate of 7 L/min. Chromatograms were recorded at a wavelength of 240 nm, and data acquisition was done using Agilent ChemStation software (Rev B.04.02 SP1, Palo Alto, CA, USA). The analysis was conducted in triplicate.

##### Total Folate Content (TF)

Folate content was analyzed by HPLC-DAD-ESI-MS assay, according to the method of Igual et al., 2021 [1]. For sample extraction, 1 g of each sample was homogenized with 5 mL of phosphate buffer (pH = 7) and sonicated using an ultrasonic bath (Elmasonic E15H, Elma). The obtained solution was further centrifuged at 4000× *g* for 10 min at a temperature of 24 °C, filtered using a nylon filter (0.45 µm, Millipore Merck KGaA, Darmstadt, Germany), and injected into the XDB C18 Eclipse (4.5 × 150 mm, particle size 5 µm) HLPC column. The column parameters were acetonitrile:acetic acid 1% in a ratio of 20:80 (*v*/*v*), flow rate of 0.5 mL/min, and a temperature of 25 °C. For the MS fragmentation, the capillary voltage was set at 300 V, a nitrogen flow of 7 L/min, and a scanning range of 120–600 *m*/*z* in the ESI (+) mode was applied. Agilent ChemStation software (Rev B.04.02 SP1) was used for data acquisition, and chromatograms were recorded at Λ = 280 nm. A folic standard curve (y = 126.25 × −16.283, r^2^ = 0.9945) with a maximum and minimum concentration of 30 µg/mL to 1 µg/mL was used. Samples were analyzed in triplicate.

##### Total Carotenoids (TC), Total Phenols (TP), and Antioxidant Capacity (AC)

The hexane/acetone/ethanol mixture was used as the solvent for the extraction of total carotenoids (TC) before and after the in vitro digestion, according to the method described by Olives Barba et al. [59]. An UV-3100PC spectrophotometer (VWR, Leuven, Belgium) was used for the sample’s absorbance at a wavelength of 446 nm. All samples were analyzed in triplicate and expressed as mg of β-carotene/100 g of dried base sample.

Methanol was used for the samples’ extraction and the Folin–Ciocalteu method was performed for the total phenols’ analysis, according to the method described by Igual et al. [60]. Briefly, 1 g of sample was mixed with 5 mL methanol, 0.5 mL HCl 5N, 2 mM of NaF, and centrifugated at 10,000 rpm at 4 °C for 10 min using an Eppendorf centrifuge (Eppendorf, Hamburg, Germany). From the supernatant, 250 µL was mixed in a 10 ml volumetric flask with 1.25 mL of Folin–Ciocalteu reagent and stored in a darker place for 8 min. Afterward, 3.75 mL of Na_2_CO_3_ with a concentration of 7.5% was added and further stored for 120 min. The samples’ absorbance was read with an UV-visible spectrophotometer (UV-3100PC, VWR, Radnor, Philadelphia, PA, USA) at 765 nm and expressed as mg gallic acid/100 g of the sample.

The antioxidant capacity was based on the DPPH method, as described previously by Igual et al. [60]. Briefly, 1 g of sample was mixed with 5 mL methanol, centrifugated under the same conditions and from the supernatant, and then 0.1 mL was mixed with 3.9 mL DPPH (0.030 g/L) and stored for 5 min. Samples’ absorbance was read at 515 nm. All the results were analyzed in triplicate and expressed as milligram Trolox equivalents (TE) per 100 grams of dried base (mg TE/100 g _db_).

##### In Vitro Extrudates’ Digestion

The method described by Minekus et al. [61] was used for mixtures’ and extrudates’ in vitro digestibility (IVD %) based on the standardized static in vitro digestion method suitable for food (COST INFOGEST network). There were mainly four steps to follow: (1) The oral phrase consisting of mixing the sample and simulating the salivary fluid (SSF) in a ratio of 1:1 with amylase for 2 min at pH = 7. (2) The gastric phrase, which is based on the mixing the oral bolus and simulate gastric fluid (SGF) (1:1) with pepsin at pH 3 for 2 h. (3) The intestinal phrase, where intestinal fluid was simulated by mixing gastric chyme with enzymes at pH 7 for 2 h. (4) The last step, consisting of samples’ centrifugation at 2600× *g* for 30 min followed by filtration, according to the method of Uribe-Wandurraga et al. [27]. The difference between the initial mass and the undigested one (after adjusting the blank assay) divided by the initial mass and multiplied by 100 was the in vitro digestibility value, as reported by Batista et al. [62]. The obtained samples at the end of the in vitro digestion process were collected as described by Minekus et al. [61] and freeze-dried with a protease inhibitor. The equation proposed by Khouzam et al. [63] was used for the bioaccessibility value as the ratio between the concentration of the bioactive compounds in the bioaccessible fraction after in vitro digestion and the samples’ bioactive compounds before the digestion process.

#### 4.4.3. Statistical Analysis

ANOVA with a high confidence level (95%, *p* < 0.05) was applied to evaluate and compare the differences between samples. Pearson correlation was used to better emphasize the correlation between samples with Statgraphics Centurion XVII Software, version 17.2.04, (Statgraphics Technologies, Inc., The Plains, VA, USA).

## 5. Conclusions

This study showed that addition of *Rosa canina* powder and pea protein in corn extrudate manufacturing does not negatively influence the physicochemical, textural, and optical properties of the final products. Even if extrusion could lead to a depleted number of bioactive compounds, their bioaccessibility might still be improved. The TC bioaccessibility was significantly higher in RPP extrudate samples compared with R extrudates, having values of 68% to 50%, respectively. RPP contents of AA, VC, and TP bioaccessibility registered values of 38%, 59%, and 90%, respectively, whereas R extrudates recorded values of 20%, 50%, and 81%, respectively. Therefore, PP and RPP could be successfully used in corn extrudates’ manufacturing to improve their nutritional value and in vitro bioaccessibility.

## Figures and Tables

**Table 1:** Mean values (and standard deviations) of water loss (W_L_), specific mechanical energy (SME), melt pressure (P), and barrel temperatures (T_1_, T_2_) of the studied samples.

	CM	RM	RPPM
W_L_ (g_w_/g_db_)	0.274 (0.012) ^b^	1.3 (0.5) ^a^	1.01 (0.14) ^a^
SME (J/g)	1099 (45) ^b^	1309 (34) ^a^	1074 (20) ^b^
P (Pa)	83 (3) ^c^	100 (16) ^b^	112 (10) ^a^
T_1_ (°C)	195.2 (0.8) ^b^	196.9 (0.3) ^a^	197.0 (0.2) ^a^
T_2_ (°C)	86.9 (0.6) ^c^	89.0 (0.7) ^b^	90.6 (0.5) ^a^

The same letter in superscript within row indicates homogeneous groups established by ANOVA (*p* < 0.05). C: control, R: rosehip enrichment, and RPP: rosehip + pea protein enrichment. M: mixtures.

**Table 2:** Mean values (and standard deviations) of extrudate characteristics.

	CE	RE	RPPE
x_w_ (g_w_/100 g)	3.45 (0.07) ^a^	1.7 (0.2) ^b^	1.866 (0.102) ^b^
a_w_	0.358 (0.003) ^a^	0.369 (0.003) ^b^	0.352 (0.003) ^d^
WAI	3.14 (0.14) ^b^	4.96 (0.16) ^a^	5.19 (0.14) ^a^
WSI (%)	22.9 (0.5) ^a^	19.0 (0.8) ^b^	9.4 (0.8) ^c^
SWE (mL_swollen_/g_dry solid_)	2.08 (0.07) ^b^	2.6 (0.3) ^ab^	2.9 (0.2) ^a^
Hy (g_w_/100 g_dry solid_)	23.0 (0.3) ^b^	26.3 (0.9) ^a^	24.3 (0.9) ^b^
SEI	13.7 (0.8) ^a^	12.9 (0.8) ^b^	10.6 (0.8) ^c^
ρ_b_ (g/cm^3^)	0.0873 (0.0009) ^a^	0.0784 (0.0004) ^c^	0.0838 (0.0008) ^b^
ε (%)	93.2 (0.2) ^a^	92.94 (0.06) ^a^	91.63 (0.04) ^b^
W_c_ (N*mm)	0.25 (0.03) ^a^	0.083 (0.019) ^c^	0.14 (0.02) ^b^
N_sr_ (mm^−1^)	10.15 (1.08) ^b^	13.54 (1.13) ^a^	14.2 (0.6) ^a^
F_s_ (N)	2.5 (0.2) ^a^	1.1 (0.3) ^c^	2.0 (0.3) ^b^
F_p_ (N)	2.01 (0.13) ^a^	0.9 (0.3) ^c^	1.7 (0.2) ^b^
N_0_	109 (11) ^b^	135 (8) ^a^	134 (8) ^a^

The same letter in superscript within row indicates homogeneous groups established by ANOVA (*p* < 0.05). C: control, R: rosehip enrichment and RPP: rosehip + pea protein enrichment, E: extrudates. Water content (x_w_), water activity (a_w_), water absorption index (WAI), water solubility index (WSI), swelling index (SWE), hygroscopicity (Hy), expansion index (SEI), bulk density (ρ_b_), porosity (ε), crispness work (W_c_), spatial frequency of structural ruptures (N_sr_), average specific force of structural ruptures (F_s_), average puncturing force (F_p_), and number of peaks (N_0_); control extrudate (CE), rosehip extrudate (RE), and rosehip + pea protein enrichment (RPPE).

**Table 3:** Mean values (and standard deviations) of color coordinates (L*, a*, b*, C*, and h*) and total color differences (ΔE) of the corn mixtures and extrudates.

	Mixtures			Extrudates		
	CM	RM	RPPM	CE	RE	RPPE
L*	81.9 (0.2) ^aA^	72.9 (0.3) ^bA^	72.6 (0.3) ^bA^	50 (4) ^aB^	50 (2) ^aB^	52 (4) ^aB^
a*	5.07 (0.12) ^cA^	14.7 (0.4) ^aA^	11.9 (0.5) ^bA^	0.7 (0.4) ^bB^	6.9 (0.3) ^aB^	6.6 (0.9) ^aB^
b*	36.6 (1.4) ^cA^	39.3 (0.4) ^bA^	40.8 (0.6) ^aA^	16.07 (1.13) ^cB^	21.7 (0.9) ^aB^	20.2 (0.8) ^bB^
C*	37.0 (1.4) ^bA^	42.0 (0.5) ^aA^	42.5 (0.7) ^aA^	16.07 (1.14) ^bB^	22.80 (0.12) ^aB^	21.3 (0.9) ^aB^
h*	82.1 (0.2) ^aB^	69.5 (0.3) ^cB^	73.8 (0.4) ^bA^	87.7 (1.4) ^aA^	72.4 (0.3) ^bA^	72 (2) ^bA^
ΔE_1_	-	13.5 (0.6) ^aA^	12.3 (0.7) ^bA^	-	8.72 (1.02) ^aB^	8.5 (1.7) ^aB^
ΔE_2_	-	-	-	39 (3) ^a^	30 (2) ^b^	30 (3) ^b^

For each parameter, the same superscript small letter within rows indicates homogeneous groups established using ANOVA (*p* < 0.05) compared to samples in mixtures or extrudates. For each sample and parameter, the same capital letter in superscript within rows indicate homogeneous groups established using ANOVA (*p* < 0.05) compared to mixtures and extrudates. Samples were mixtures (M) and extrudates (E). C: control, R: rosehip enrichment, and RPP: rosehip + pea protein enrichment. L* (lightness), a* (red/green coordinate), b* (yellow/blue coordinate), C* (chroma), and h* (tone).

**Table 4:** Mean values (and standard deviations) of corn mixtures and extrudates’ component content and extrusion loss.

	Mixtures			Extrudates			Extrusion Loss (%)		
	CM	RM	RPPM	CE	RE	RPPE	CE	RE	RPPE
AA	5.576 (0.005) ^cA^	24.231 (0.008) ^bA^	30.637 (0.005) ^aA^	4.506 (0.007) ^cB^	12.50 (0.03) ^aB^	7.40 (0.02) ^bB^	19.19 (0.05)	48.42 (0.09) ^b^	75.84 (0.07) ^a^
VC	20.47 (0.05) ^cA^	45.37 (0.05) ^bA^	56.81 (0.04) ^aA^	16.81 (0.04) ^cB^	35.67 (0.08) ^aB^	33.62 (0.03) ^bB^	17.866 (0.002) ^c^	21.39 (0.09) ^b^	40.818 (0.007) ^a^
FT	0.073 (0.004) ^cA^	0.703 (0.005) ^aA^	0.61 (0.02) ^bA^	0.068 (0.004) ^cB^	0.383 (0.006) ^bB^	0.506 (0.007) ^aB^	6.9 (0.5) ^c^	45.5 (0.4) ^a^	16.58 (1.05) ^b^
TC	3.75 (0.09) ^cA^	43.74 (0.14) ^aA^	22.73 (0.03) ^bA^	2.11 (0.06) ^cB^	7.85 (0.04) ^aB^	5.76 (0.02) ^bB^	44 (3) ^c^	82.05 (0.14) ^a^	74.68 (0.12) ^b^
TP	23.68 (1.15) ^cA^	334 (9) ^aA^	272 (3) ^bA^	14.4 (0.3) ^bB^	169.2 (0.7) ^aB^	169.3 (0.8) ^aB^	39 (2) ^b^	49.7 (1.5) ^a^	37.8 (0.5) ^b^
AC	1.73 (0.14) ^cA^	201 (2) ^aA^	64.7 (0.3) ^bA^	n.d. ^bB^	14.01 (0.13) ^aB^	12.3 (1.3) ^aB^	100 (0) ^a^	93.06 (0.6) ^b^	81 (2) ^c^

For each sample and parameter, the same capital letter in superscript within the row indicates homogeneous groups established by ANOVA (*p* < 0.05) comparing mixtures and extrudates; for each parameter, the same superscript small letter within rows indicates homogeneous groups established using ANOVA (*p* < 0.05) compared to samples in mixtures, extrudates and extrusion loss (%). Samples were mixtures (M) and extrudates (E); C: control, R: rosehip enrichment, and RPP: rosehip + pea protein enrichment. Ascorbic acid (AA), vitamin C (VC), folates (FT), total carotenoids (TC), total phenols (TP) content (mg/100 g_dry weight_), and antioxidant capacity (AC) (mg TE/100 g_dry weight_).

**Table 5:** Mean values (and standard deviations) of corn mixtures’ and extrudates’ component content after in vitro digestion.

	Mixtures			Extrudates		
	CM	RM	RPPM	CE	RE	RPPE
AA	2.186 (0.005) ^cB^	4.346 (0.002) ^aA^	2.1429 (0.0012) ^bB^	2.511 (0.009) ^cA^	2.669 (0.003) ^bB^	2.751 (0.003) ^aA^
VC	11.70 (0.02) ^cA^	18.70 (0.04) ^aA^	18.38 (0.03) ^bB^	11.78 (0.02) ^cA^	17.555 (0.014) bB	19.22 (0.03) ^aA^
FT	0.0356 (0.0002) ^cA^	0.514 (0.002) ^aA^	0.458 (0.004) ^bA^	0.0336 (0.0002) ^cB^	0.311 (0.007) ^bB^	0.446 (0.002) ^aB^
TC	0.62 (0.04) ^cA^	4.99 (0.03) ^aA^	4.43 (0.12) ^bA^	0.64 (0.09) ^bA^	3.85 (0.09) ^aB^	3.82 (0.03) ^aB^
TP	6 (2) cA	180.6 (0.7) ^aA^	155 (8) ^bA^	6.4 (0.3) ^bA^	132 (4) ^aB^	128 (5) ^aB^
AC	0.89 (0.09) ^cA^	104.5 (1.4) ^aA^	44 (2) ^bA^	n.d. ^bB^	9.7 (0.8) ^aB^	9.7 (0.2) ^aB^

For each sample and parameter, the same capital letter in superscript within the row indicates homogeneous groups established by ANOVA (*p* < 0.05) comparing mixtures and extrudates; for each parameter, the same superscript small letter within rows indicates homogeneous groups established using ANOVA (*p* < 0.05) compared to samples in mixtures and extrudates. Samples were mixtures (M) and extrudates (E); C: control, R: rosehip enrichment, and RPP: rosehip + pea protein enrichment. Ascorbic acid (AA), vitamin C (VC), folates (FT), total carotenoids (TC), total phenols (TP) content (mg/100 g), and antioxidant capacity (AC) (mg TE/100 g).

## Data Availability

Not applicable.
